# Cardioprotection vs. regeneration: the case of extracellular vesicle-derived microRNAs

**DOI:** 10.1007/s00395-021-00857-9

**Published:** 2021-03-19

**Authors:** Kai C. Wollert, Loren J. Field

**Affiliations:** 1 Division of Molecular and Translational Cardiology, Department of Cardiology and Angiology, Hannover Medical School, Carl-Neuberg-Straße 1, 30625 Hannover, Germany; 2 Krannert Institute of Cardiology and the Herman B. Wells Center for Pediatric Research, Indiana University School of Medicine, 1044 W. Walnut Street, Indianapolis, IN 46202 USA

Cardiomyocyte renewal in the adult mammalian heart occurs via proliferation of pre-existing cardiomyocytes, as opposed to differentiation from endogenous cardiomyogenic stem cells [7]. Unfortunately, the intrinsic renewal capacity of adult mammalian cardiomyocytes is limited, with similarly low rates reported for rodents and humans [3, 17]. Consequently, injuries associated with massive cardiomyocyte death, like an acute myocardial infarction (MI), promote scarring as opposed to noticeable tissue regeneration [14]. Stimulating cardiomyocyte proliferation may, however, provide a means to achieve myocardial regeneration after injury [2, 5, 6, 8, 13].

In this issue of *Basic Research in Cardiology*, Jung and colleagues propose a new microRNA-based strategy for ischemic heart repair [10]. They initially observed that human-induced pluripotent stem cell-derived cardiomyocytes (iCMs) release large amounts of extracellular vesicles (EVs) when cultured under hypoxic conditions. EVs are membrane-enclosed nanoscale particles that can transfer protein, lipid, RNA, and DNA cargo between cells through the extracellular space [12]. Three microRNAs (miR-20b, miR-92a, and miR-363), originating from a common pre-microRNA transcript, were highly enriched in hypoxic iCM-derived EVs. Treatment with iCM-derived EVs or directly overexpressing the three miRs protected iCMs from hypoxia-induced cell death. Conversely, downregulating the three miRs reduced iCM survival under hypoxic conditions, thus revealing a cell-autonomous protective mechanism [10].

Exploring therapeutic potential, the authors turned to a mouse model of acute MI induced by permanent coronary artery ligation. Injecting EVs or miR-20b-, miR-92a-, and miR-363-mimics encapsulated in a biodegradable polymer into the infarct border zone immediately after MI attenuated left ventricular dilatation and systolic dysfunction as assessed by magnetic resonance imaging (MRI) at 2 and 4 weeks. At 4 weeks, miR-treated mice had developed only very small scars (encompassing ~ 13% of the LV myocardium) compared with control animals (~ 38%). Mechanistically, the authors propose that the EVs, specifically the three miRs, stimulated cardiomyocyte proliferation in the infarct border zone leading to endogenous self-repair of the heart [10]. But is this a plausible explanation?

Supporting their case, the authors found that overexpressing the three miRs in hypoxic iCMs induced mRNA signatures related to cell cycle G1/S transition, DNA replication, and G2/M phase. As evidenced by EdU incorporation and Ki67 and Aurora B expression, these gene expression changes were associated with a modest increase in the number of iCMs progressing through the cell cycle. Using bioinformatic tools and miR-target validation, the authors identified Notch3 as a direct downstream target of the three miRs. Indeed, selectively targeting Notch3 using a small interfering RNA mimicked the effects of miR-overexpression in iCMs. Considering that iCMs provide a suitable platform to study cardiomyocyte proliferation in vitro [4], the authors regrettably do not report the effects of miR-transfection or Notch3 targeting on iCM cell numbers [10].

Quantitation of cell cycle and myofiber marker colocalization via confocal imaging of tissue sections suggested a high rate of cardiomyocyte cell cycle activity in miR-treated infarcted mice [10]. However, this approach is prone to misidentification of cardiomyocyte nuclei when employed in the absence of a cardiomyocyte-restricted nuclear marker or a cell membrane marker [1], as was the case here. Because miR-treatment also appeared to increase cell cycle activity in non-cardiomyocytes, nuclear misidentification would erroneously suggest increased cell cycle activity in the cardiomyocyte compartment of treated animals [18]. It was surprising that miR-mimics would elicit a similar induction of cell cycle activity in in vivo adult cardiomyocytes (which are exceedingly reluctant to proliferate) [3, 17] as compared to iCMs (a cell population which is highly responsive to mitogenic stimuli) [4]. It was also rather surprising that a mitotic marker would exhibit a larger labeling index than an S-phase marker [10], given that S-phase duration is much longer than M-phase duration [16]. Finally, no direct proof of cardiomyocyte cytokinesis is provided in the in vivo studies.

Permanent coronary artery ligation results in transmural necrosis, affecting cardiomyocytes and non-parenchymal cells alike. MRI suggested that the infarcted area was replaced by full-thickness, viable myocardium already 2 weeks after EV delivery. To curtail scar formation within this time frame would require exceptionally high rates of cardiomyocyte proliferation. Notably, miR-mimics were taken up not only by cardiomyocytes, but also vascular cells and fibroblasts in the infarct border zone. Microvessel density in the border zone was ~ 30% greater in miR-treated mice at 4 weeks [10]. Although, with small animal numbers, this potential treatment effect did not reach statistical significance, therapies promoting comparable improvements in border zone capillarization may very well reduce scarring after MI in mice [11, 15, 19]. MiR-mimics may have also reduced cell death after MI as suggested by their effects on hypoxic iCMs in vitro and their impact on the number of caspase 3^+^ cells in the infarct border zone in vivo [10]. Serial histopathological examinations early after EV or miR-delivery would have been critical to understand the sequence of events and relative contributions of myocardial salvage, improved wound healing, and cardiomyocyte proliferation [5].

While the authors should be congratulated on defining a novel therapeutic approach to infarct repair, more evidence is needed to convince us, beyond a reasonable doubt, that miR-20b-, miR-92a-, and miR-363-mimics mediate their salubrious effects by stimulating myocardial regeneration, as opposed to promoting cardioprotection and wound healing [9, 14, 19].
